# Adaptation and validation of the evidence‐based practice beliefs and implementation scales into German

**DOI:** 10.1002/nop2.593

**Published:** 2020-08-26

**Authors:** Emmanuelle Kerwien‐Jacquier, Henk Verloo, Filipa Pereira, Karin Anne Peter

**Affiliations:** ^1^ School of Health Sciences HES‐SO Valais/Wallis University of Applied Sciences and Arts of Western Switzerland Visp Switzerland; ^2^ Division of Applied Research & Development in Nursing Bern University of Applied Sciences Bern Switzerland

**Keywords:** acute hospital, EBP beliefs, EBP implementation, evidence‐based practice, German, nurse, psychometric assessment

## Abstract

**Aims:**

To culturally adapt and translate the *Evidence‐Based Practice Belief Scale* (EBP‐B) and the *Evidence‐Based Practice Implementation Scale* (EBP‐I), explore the psychometric properties of their validated German versions and compare results with those of the original scales.

**Design:**

Cross‐sectional descriptive study.

**Method:**

The study was conducted on a sample of 131 Registered Nurses in a Swiss German hospital. Internal consistency was rated using Cronbach's alpha. Principal component analysis using varimax rotation was used to determine construct validity. The study was undertaken in accordance with the STROBE‐checklist in Appendix S1.

**Results:**

German versions of the EBP‐B and EBP‐I showed good reliability. Their Cronbach alphas showed lower values than those of the original scales. Principal component analysis showed medium‐to‐high factor loading. Principal component analyses using varimax rotations of the EBP‐B's 16 items and the EBP‐I's 17 items resulted in four‐factor and five‐factor solutions, respectively.

**What does this paper contribute to the wider global clinical community?**

- The German EBP‐B and EBP‐I scales are valid and showed good reliability (EBP‐B = 0.85; EBP‐I = 0.88) and medium‐to‐high factor loading (EBP‐B > 0.4; EBP‐I > 0.4);
- The German EBP‐B and EBP‐I scales make it possible to measure the attitude towards EBP and the extent to which EBP is implemented in the everyday working life in German‐speaking countries;
- These scales can be used to assess the effectiveness of organizational EBP strategies.

## 1 INTRODUCTION

Increasing numbers of healthcare institutions recognize that the use of evidence‐based practice (EBP) is a high‐level skill which helps to ensure optimal, effective, safe and cost‐efficient care (Orta et al., 2016). EBP processes use several sources of information: documentary research evidence in the literature, healthcare professionals' clinical expertise and experience in precise contexts, and patient preferences (Mackey & Bassendowski, 2017). Sackett, Rosenberg, Gray, and Haynes und Richardson (1996) defined EBP as medicine based on conclusive results, which involves the conscientious, explicit and judicious use of the best scientific proof in decision‐making about patients (Sackett et al., 1996).

Evidence‐based practice is by no means an optional activity for nurses; it is an integral part of their daily interactions with patients (Melnyk, 2007). Its relevance has encouraged the development of specialized centres for its promotion, through a variety of activities, and its teaching as part of the primary nursing curriculum (Dotson et al., 2015).

The increase in multinational and multicultural research projects necessitates the adaptation and psychometric validation of auto‐administered questionnaires for use in languages other than their source language. Most questionnaires have been developed in English‐speaking countries, but even within these countries, researchers have to consider their significant immigrant populations: in studies of beliefs and attitudes about health care, especially, their exclusion could lead to a systematic bias in studies of healthcare utilization (Beaton, Bombardier, Guillemin, & Ferraz, 2000). Adapting a self‐administered questionnaire for use in a new country, culture or language requires the use of a unique, evidence‐based methodology to ensure equivalence between the source questionnaire and its new versions. It is recognized that if measurement instruments are to be used across cultures, then their items must not only be well translated, but they must also be adapted culturally in order to maintain the validity of their content at a conceptual level. Attention to this level of detail in translated instruments gives researchers greater confidence in the results of multinational trials or outcome evaluations. The term *cross‐cultural adaptation* encompasses a process which looks at issues of both language (translation) and cultural adaptation during the process of preparing a questionnaire for use in another setting. The process of cross‐cultural adaptation tries to produce equivalency between source and target based on content. It is sometimes assumed that this process is enough to ensure that psychometric properties like validity and reliability will be retained at the item or scale level. However, this is not necessarily the case. If the new culture has a different way of approaching a task—one that makes it inherently more or less problematic compared to other items—then this would change the validity, especially in terms of any item‐level analysis (Kalfoss, 2019).

## 2 BACKGROUND

According to the International Council of Nurses, nurses should carry out their professional activities in accordance with best practices based on scientific evidence and work to ensure their professional development (International Council of Nurses, 2019). EBP is today considered a key strategy for improving the quality of care and boosting professional job satisfaction. Moreover, using EBP helps to cut healthcare costs (Johansson, Fogelberg‐Dahm, & Wadensten, 2010; Melnyk et al., 2018).

Although it is now well recognized that EBP improves the quality of care and patient treatment outcomes, the implementation and maintenance of an institutional culture of EBP remains a real challenge in many healthcare systems (Melnyk & Fineout‐Overholt, 2011; Warren, Montgomery, & Friedmann, 2016). Even if nurses had positive attitudes towards EBP, they only made limited use of it in their work (Cruz et al., 2016; Stokke, Olsen, Espehaug, & Nortvedt, 2014; Zelenikova et al., 2016).

Several factors may play a part in blocking the implementation of EBP, including contextual issues (Duffy, Culp, Sand‐Jecklin, Stroupe, & Lucke‐Wold, 2016) and organizational shortcomings (Brown et al., 2010; Duncombe, 2018; Johnston et al., 2016). Furthermore, the age, the number of years of professional experience, postgraduate training and social skills all influence the use of new sources of knowledge and the perception of obstacles to the implementation of EBP (Baird & Miller, 2015; Dalheim, Harthug, Nilsen, & Nortvedt, 2012; Shin & Lee, 2017). A positive attitude towards EBP is a prerequisite to its implementation; it helps to encourage other nurses in the healthcare facility and to create a culture of EBP within the establishment (Bonner & Sando, 2008; Melnyk et al., 2004; Stokke et al., 2014). The *Advancing Research and Clinical practice through close Collaboration* (ARCC) model includes key strategies for individual and organizational change in the context of applying Best Practice. The ARCC helps healthcare professionals to acquire the latest knowledge, attitudes and competencies in EBP, thus enabling consistent implementation and the development of an EBP‐based culture—a culture which will ensure the use of Best Practice and the achievement of high‐quality outcomes. The ARCC model describes the strategy for implementing and maintaining EBP in healthcare institutions (Melnyk, Fineout‐Overholt, Giggleman, & Choy, 2017).

A recent literature research revealed that there are no current studies in this topic in German‐Speaking Switzerland. The question of a suitable measuring instrument also arises here. The *EBP Belief Scale* (EBP‐B) and the *EBP Implementation Scale* (EBP‐I) by Melnyk, Fineout‐Overholt, Mays, (2008) are suitable scale and are a part of the ARCC model. A German validated scale translation is missing. Therefore, the present study's goals were to determine the psychometric properties of the EBP‐B and EBP‐I scales in German and compare their results against those of the original scales.

## 3 METHOD

### 3.1 Design

This cross‐sectional descriptive study examined the internal consistency and conceptual validity of the German‐language translations of the EBP‐B and EBP‐I scales based on a convenience sample of participants. The study was undertaken in accordance with the STROBE‐checklist in Appendix S1.

### 3.2 Research population and sample size

The research population was recruited from a university hospital in Switzerland. The hospital's directorate of nursing care agreed to its personnel's participation because no personal medical data were required. The criterion for inclusion was holding a Swiss nursing diploma from a specialized school or a university of applied sciences, or a foreign diploma recognized as equivalent. The exclusion criteria ruled out temporary nurses, pool nurses and nurses who had only been working in the participating acute care department for 3 months or less. Nurses who had participated in the EBP scale testing were also excluded.

The sample size was chosen to match the samples used to test the psychometric properties of the two scales in other languages (between *N* = 91–*N* = 471) (Thorsteinsson, 2012; Verloo, Desmedt, & Morin, 2017; Wang et al., 2012; Zelenikova et al., 2016). Previous literature indicates that a good Cronbach's alpha of >0.8 is achieved with approximately 90 participants (Zelenikova et al., 2016). Therefore, our target study sample was at least 90 participants.

**FIGURE 1:**
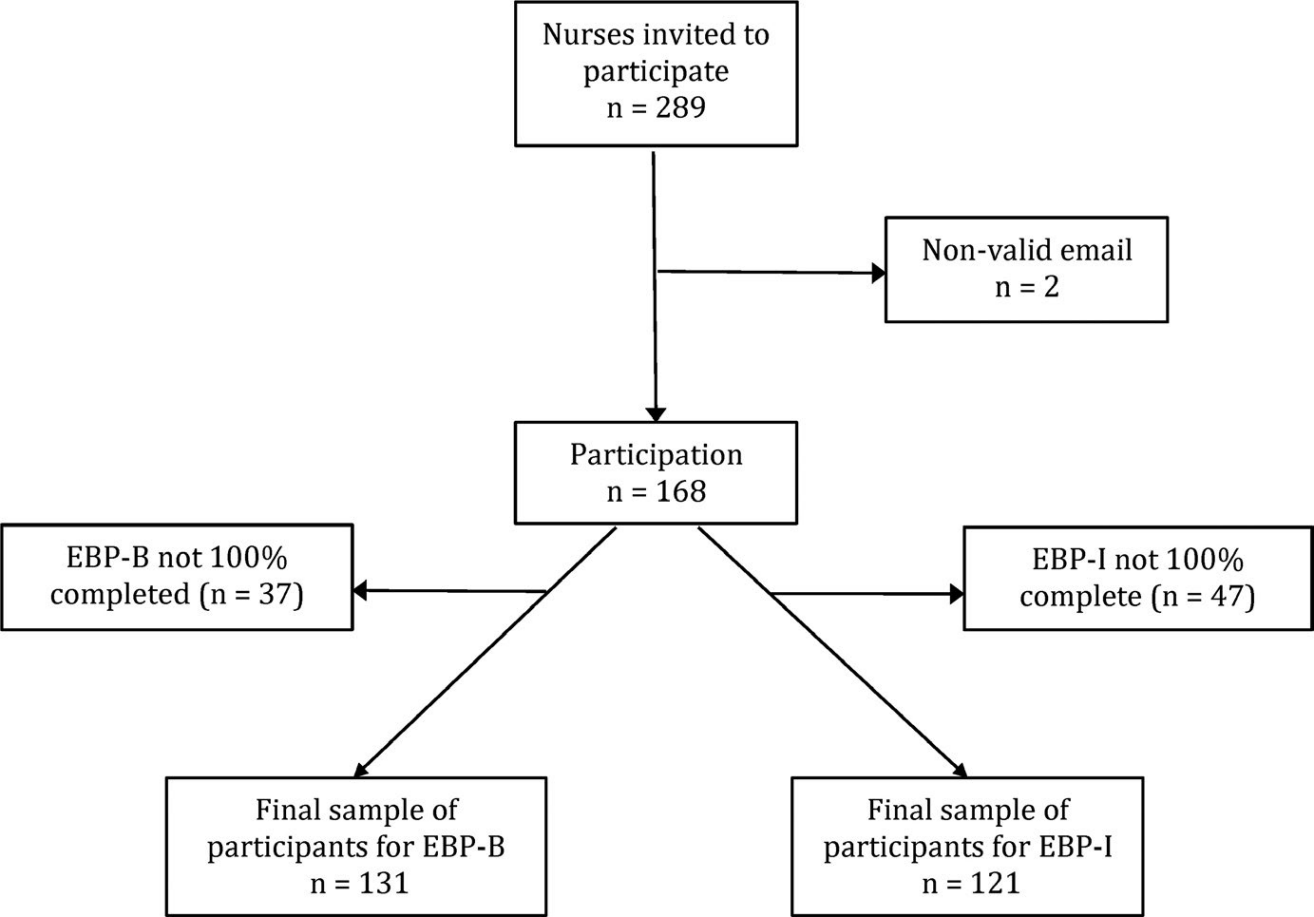
Recruitment and participation flow chart

The final sample of participants consisted of 131 nurses. Figure 1 presents more detail on the response rate of participants and on the final sample for each scale (EBP‐B and EBP‐I).

### 3.3 Data collection

Data collection took place between November 2018–March 2019. A total of 289 questionnaires were sent out to eligible participants by email. They were sent reminder emails after 2, 4 and 8 weeks. Questionnaires were accompanied by written information on the study's objectives, the conditions of participation and data confidentiality. Responding to the questionnaire was taken as written informed consent to participate as participation was anonymous.

### 3.4 Instruments

#### 3.4.1 The original scales

The EBP‐B and EBP‐I scales developed by Melnyk et al. (2008) were used to measure attitudes towards EBP and how the target population implemented EBP in their daily clinical practice. After authorization from the authors of the original scales (Melnyk et al., 2008), the German‐language versions were considered to be new instruments and therefore were subjected to the same psychometric testing used on the originals (Melnyk et al., 2008).

The EBP‐B contains 16 items and investigates respondents' attitudes towards EBP. Each item is answered on a five‐point Likert scale ranging from 1 (strongly disagree)–5 (strongly agree). The sum of the 16‐item responses was calculated after inversing the scores for two items formulated negatively: “I believe EBP is difficult” and “I believe that EBP takes too much time.” The resulting total scores could range between 16 (minimum)–80 (maximum). Each item of the original EBP‐B scale presented a factor loading >0.35 and a high Cronbach's alpha factor of 0.90. Using a varimax rotation, Melnyk et al. measured a single construct (Melnyk et al., 2008).

The EBP‐I contains 18 items and records the frequency with which participants have used EBP over the last 8 weeks. Items are evaluated on a five‐point Likert scale ranging from 0 (0 times)–4 (8 or more times). The resulting total score of the sum of the 18 items could range between 0 (minimum)–72 (maximum). For the original EBP‐I scale, Melnyk et al. found a factor loading >0.60 for the original EBP‐I scale, together with and a Cronbach's alpha of 0.96; using varimax rotation, they computed a single construct (Melnyk et al., 2008).

#### 3.4.2 Cultural adaptation and translation of evidence‐based practice scales into German

Appropriate and psychometrically validated instruments are essential for making comparisons between studies. The EBP‐B and EBP‐I scales developed by Melnyk et al. (2008) have been translated and psychometrically validated in the French (Verloo et al., 2017), Norwegian (Stokke et al., 2014), Icelandic (Thorsteinsson, 2012), Slovak and Czech (Zelenikova et al., 2016) and Chinese languages (Wang et al., 2012).

Translation into German and cultural adaptations was guided by the ten stages recommended by Wild et al. (2005). Three items on the EBP‐B scale had to be improved after checking their back‐translation against the original scales and discussing them with their author as the differences seemed significant. The EBP‐I item “Accessed the National Guidelines Clearinghouse” was removed because no comparable database exists in Switzerland. After five cognitive debriefings with nursing professionals with differing levels of training, some items were reformulated more precisely; this clarified and improved their overall comprehension of the questions.

A pilot test of the scales' final versions was carried out with 11 nursing professionals with similar sociodemographic and professional characteristics to the eligible participants. This step enabled us to make final adjustments to the questionnaires' instructions and presentation. In addition to the scales, participants were also sent a questionnaire on their sociodemographic and professional characteristics. Some knowledge of the concept of EBP was desirable, but it was not a necessary condition for completing the scales (Melnyk & Fineout‐Overholt, 2011).

### 3.5 Data analysis

Data analysis was conducted using SPSS 25^®^. The sum score for the EBP‐B and EBP‐I was calculated according to Melnyk et al. (2008), and all cases with missing values were excluded. Considering reverse‐scored items, “I believe EBP is difficult” and “I believe that EBP takes too much time,” were recoded before analysis to avoid errors in the total score.

In a first step, mean, standard deviation (*SD*), median and interquartile ranges were calculated for each item using descriptive analysis. The normality of the data distributions was evaluated using the Kolmogorov–Smirnov test (Field, 2013). Also, the responses' floor and ceiling effects were analysed using the cut‐off of 20% in the positive or negative skewness of the distribution in relation to each item's mean (Wang, Zhang, McArdle, & Salthouse, 2008).

In a second step, an exploratory factor analysis (EFA) was conducted to establish the construct validity of the German‐language versions. We used a Bartlett test to determine whether correlations were significantly different from zero (*p* < .05) (Field, 2013) and the Kaiser–Meyer–Olkin (KMO) coefficient to indicate whether the data were suitable for factorial analysis (Field, 2013; Kaiser, 1974). Although a principal component analysis (PCA) is not strictly speaking a factor analysis (Field, 2013), the conceptual validities of the two scales could be determined more precisely by using PCA with varimax rotation, according to Melnyk et al. (2008). The calculations enabled us to verify whether the translated versions of the EBP‐B and the EBP‐I shared the same factors as those in Melnyk's study. The validities of the two studies were analysed separately because they used different semantic and numerical scales for their different objectives, which ruled out combining them in a single factor analysis (Melnyk et al., 2008). We also examined the eigenvalue criterion (Kaiser, 1960) and the scree plot (Cattell, 1966). To preserve factor independence, we used a varimax rotation which resulted in non‐correlated factors (Field, 2013). For factor interpretation, elements whose factor loading after varimax rotation was less than 0.30 were excluded (Field, 2013).

In a third step, internal consistencies of the instruments and their subscales were determined using Cronbach's alpha (desired values >0.80).

## 4 RESULTS

### 4.1 Sample description

A total of 73% of participants (*n* = 88) were holders of tertiary‐level nursing diplomas. Participants had mean 17.9 years of professional experience in nursing care (*SD* 10.6) and mean 8.4 years of experience in current professional post (*SD* 8.0). Participant had a mean age of 39 years old (*SD* 11.1); most participant (92.2%) were women and aware of the concept of EBP (84.5%). Table 1 shows the participants' sociographic and professional data.

**TABLE 1:** Participants' sociographic and professional data (*N* = 131)

	Mean	Min/Max	*SD*
Age (*n* = 131)	39.2	23/64	11.1
Years of experience in current professional post (*n* = 121)	8.4	0.3/32	8.0
Years of experience in nursing care (*n* = 121)	17.9	0.3/43	10.6
Rate of full‐time equivalent work (*n* = 121)	75.7	20/100	23.5

	Participants	%
Sex (*n* = 129)		
Female	119	92.2
Male	10	7.8
Country where nursing diploma was obtained (*n* = 120)		
Switzerland	109	90.8
Other	11	9.2
Level of nursing education (*n* = 120)		
Tertiary	88	73.3
Bachelor's degree	23	19.2
Master's degree	8	6.7
PhD	1	0.8
Professional post held^a^ (*n* = 121)		
Registered Nurse	93	65.5
Clinical nurse specialist	21	14.7
Management or administrative function	20	14.1
Other	8	5.6

^a^ More than one response was possible.

### 4.2 Psychometric properties of the EBP‐B scale

The results of the Bartlett test (*χ* ^2^ = 784.140, *p* < .000) and the KMO test (0.797) for the EBP‐B scale indicated that the variables were suitable for undergoing a factor analysis.

#### 4.2.1 EBP‐B scale validity

**FIGURE A1:**
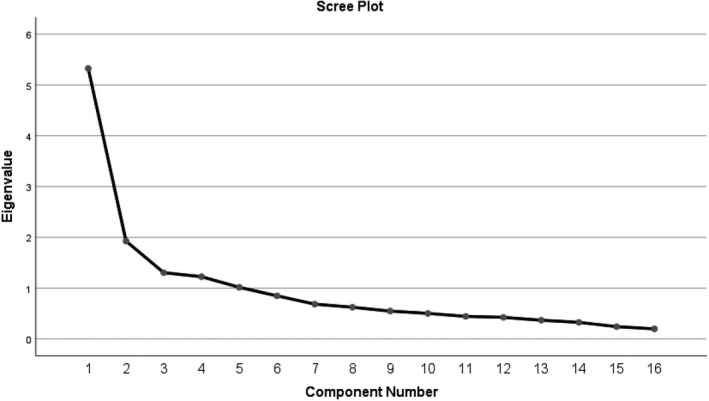
Scree plot of the factor analysis of the EBP‐B scale

A PCA assessed the underlying structure of the 16‐item German‐language version of the EBP‐B scale (Table 2). Figure A1 presents the Scree plot of the factor analysis of the EBP‐B scale. A varimax rotation using Kaiser normalization found five‐factor subscales, not exactly matching the index of four subscales in the original EBP‐B scale. The factors explained 67.47% of the variance. All the items had a factor loading after varimax rotation of >0.458. Seven items had cross‐loading. The factors analysed were as follows: *value beliefs, implementation beliefs, knowledge beliefs, time and difficulty beliefs* and *effective evidence‐based care beliefs*. The first factor presented an eigenvalue of 5.325, accounting for 33.28% of the variance in the scale. The four other factors had eigenvalues >1.0 (1.926, 1.303, 1.225 and 1.016, respectively), accounting for 12.04%, 8.15%, 7.66% and 6.35% of the scale's variance, respectively. The item “I believe the care that I deliver is evidence‐based” is the only one included in factor five.

**TABLE 2:** Factor loading for the principal component analysis using varimax rotation for the EBP‐B scale (*n* = 131)

Items in the EBP scale	Factor				
	1	2	3	4	5
I am sure that evidence‐based guidelines can improve clinical care	0.839	0.184	−0.063	0.017	0.039
I am sure that implementing EBP will improve the care that I deliver to my patients	0.756	0.358	0.025	0.073	0.177
I believe that critically appraising evidence is an important step in the EBP process	0.725	0.150	0.118	0.208	−0.077
I believe that EBP results in the best clinical care for patients	0.718	−0.034	0.263	0.135	0.001
I am sure that I can implement EBP	0.458	0.407	0.431	0.126	−0.049
I believe that I can overcome barriers to implementing EBP	0.244	0.779	0.055	−0.021	−0.201
I am sure that I can implement EBP in a time‐efficient way	0.333	0.762	0.064	0.125	−0.030
I am confident about my ability to implement EBP where I work	0.094	0.710	0.155	0.090	0.229
I believe that I can search for the best evidence to answer clinical questions in a time‐efficient way	0.089	0.573	0.379	0.180	0.040
I am sure that I can access the best resources to implement EBP	−0.118	0.461	0.370	0.327	0.125
I am clear about the steps of EBP	0.253	0.061	0.849	−0.077	−0.103
I am sure about how to measure the outcomes of clinical care	0.005	0.143	0.781	0.098	0.163
I know how to implement EBP sufficiently enough to make practice changes	0.074	0.345	0.622	0.261	0.231
I believe EBP is difficult (reverse‐scored)	0.084	0.151	0.203	0.822	−0.097
I believe that EBP takes too much time (reverse‐scored)	0.312	0.094	−0.036	0.793	0.053
I believe the care that I deliver is evidence‐based	0.058	0.021	0.134	−0.041	0.936
Eigenvalues	5.325	1.926	1.303	1.225	1.016
% of variance	33.28	12.04	8.15	7.66	6.35
Explained total variance	67.47%				

#### 4.2.2 EBP‐B scale reliability

Table 3 presents the means, SDs and skewness distribution of the scores for each item on the EBP‐B scale. The highest rated items concerned beliefs about the positive effects of EBP. The lowest rated items concerned the individual's capacity to implement EBP. Except for the item “I believe the care that I deliver is evidence‐based,” all the items in the German‐language version were inter‐correlated with at least one other item, at 0.30. A ceiling effect was revealed among the items “I am sure that implementing EBP will improve the care that I deliver to my patients,” “I believe the care that I deliver is evidence‐based” and “I believe that I can overcome barriers in implementing EBP” with a positive skewness distribution of >20% (Table 3).

**TABLE 3:** EBP‐B scale mean, *SD*, skewness distribution, median and interquartile (*n* = 131)

Items in the EBP scale	Mean	*SD*	Skewness	Median	Interquartile 75%
I am sure that evidence‐based guidelines can improve clinical care	4.35	0.64	−0.656	4.00	5.00
I believe that critically appraising evidence is an important step in the EBP process	4.26	0.67	−0.518	4.00	5.00
I believe that EBP results in the best clinical care for patients	4.18	0.64	−0.709	4.00	5.00
I am sure that implementing EBP will improve the care that I deliver to my patients	4.10	0.71	−0.928 ^a^	4.00	5.00
I believe the care that I deliver is evidence‐based^a^	3.87	0.71	−1.143	4.00	4.00
I am confident about my ability to implement EBP where I work	3.68	0.85	−0.690	4.00	4.00
I am sure that I can implement EBP	3.60	0.84	−0.650	4.00	4.00
I believe that I can overcome barriers in implementing EBP	3.60	0.86	−0.998 ^a^	4.00	4.00
I am sure that I can implement EBP in a time‐efficient way	3.44	0.94	−0.520	4.00	4.00
I am clear about the steps of EBP	3.35	1.04	−0.413	4.00	4.00
I believe that I can search for the best evidence to answer clinical questions in a time‐efficient way	3.33	0.96	−0.167	4.00	4.00
I believe EBP is difficult. (reverse‐scored)	3.18	1.04	−0.152	3.00	4.00
I believe that EBP takes too much time. (reverse‐scored)	3.13	1.14	−0.164	3.00	4.00
I am sure about how to measure the outcomes of clinical care	3.11	1.14	−0.181	3.00	4.00
I am sure that I can access the best resources to implement EBP	3.06	0.97	0.028	3.00	4.00
I know how to implement EBP sufficiently enough to make practice changes	3.04	1.05	−0.117	3.00	4.00
EBP‐B scale	57.27	8.08		58.00	63.00

^a^ Ceiling effect of the item.

Cronbach's alphas were computed and summed to assess whether the data from the 16 items in the German‐language version of the EBP‐B formed a reliable scale. The overall alpha was 0.853 (95% CI = 0.813, 0.887), indicating that the scale had very good internal consistency. Cronbach's alphas for the subscales were as follows: *value beliefs* *α* = 0.814 (95% CI = 0.759, 0.860); *implementation beliefs* *α* = 0.770 (95% CI = 0.701, 0.827); *knowledge beliefs* *α* = 0.757 (95% CI = 0.675, 0.821); and *time and difficulty beliefs* *α* = 0.653 (95% CI = 0.509, 0.754).

### 4.3 Psychometric properties of the EBP‐I scale

The results of the Bartlett test (*χ* ^2^ = 946.142, *p* < .000) and the KMO (0.777) test for the EBP‐I scale indicated that the variables were suitable for undergoing a factor analysis.

#### 4.3.1 EBP‐I scale validity

Table 4 presents the means, SDs and the skewness distribution of the scores for each item on the EBP‐I scale. The highest rated item concerned the data collected about a patient problem. The lowest rated item concerned access to the Cochrane database. Except for the item “Generated a PICO question about my clinical practice,” all the items in the German‐language version were inter‐correlated with at least one other item on the scale at 0.303. All the items presented a floor effect.

**TABLE 4:** Factor loading for principal component analysis using varimax rotation for the EBP‐I scale (*n* = 121)

Items in the EBP‐I scale	Factor				
	1	2	3	4	5
Changed practice based on patient outcome data	0.886	−0.004	0.098	0.084	0.059
Evaluated a care initiative by collecting patient outcome data	0.844	0.044	−0.070	0.097	0.183
Shared the outcome data collected with colleagues	0.819	0.166	−0.067	0.096	0.065
Collected data on a patient problem	0.644	0.178	0.214	0.103	0.067
Used evidence to change my clinical practice	0.557	0.160	0.270	0.322	−0.468
Promoted the use of EBP to my colleagues	0.468	0.351	0.202	0.308	0.384
Accessed the Cochrane database of systematic reviews	0.004	0.871	0.060	0.078	0.187
Read and critically appraised a clinical research study	0.151	0.856	0.058	0.052	−0.059
Critically appraised evidence from a research study	0.174	0.844	0.152	0.097	−0.061
Shared evidence from a study in the form of report/presentation to >2 colleagues	0.052	0.664	0.126	0.344	0.280
Shared evidence from a research study with a multidisciplinary team member	0.025	0.223	0.782	−0.032	0.157
Shared evidence from a research study with a patient/family member	−0.011	−0.084	0.733	0.417	−0.052
Informally discussed evidence from a research study with a colleague	0.266	0.471	0.541	0.003	0.099
Shared an EBP guideline with a colleague	0.130	0.136	0.029	0.894	0.072
Used an EBP guideline/systematic review to change clinical practice where I work	0.351	0.236	0.184	0.608	0.011
Generated a PICO question about my clinical practice	0.109	0.188	0.370	−0.29	0.595
Evaluated the outcomes of a practice change	0.436	0.029	−0.039	0.210	0.584
Eigenvalues	5.719	2.365	1.447	1.162	1.001
% of Variance	33.64	13.91	8.51	6.84	5.89
Explained total variance	68.79%				

**FIGURE A2:**
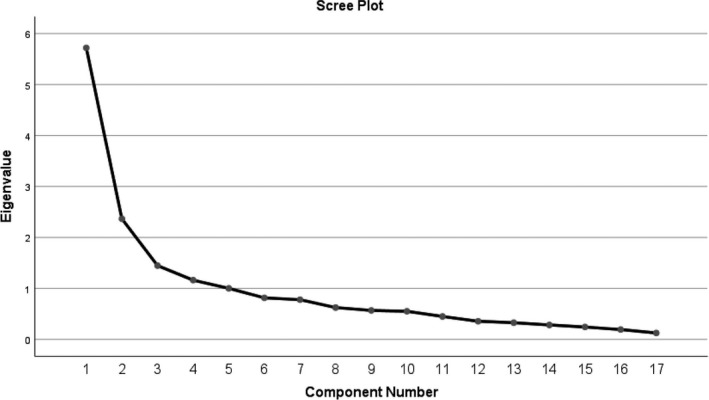
Scree plot of the factor analysis of the EBP‐I scale

A PCA assessed the underlying structure of the 17 items in the German‐language translation of the EBP‐I scale. Figure A2 presents the scree plot of the factor analysis of the EBP‐I scale. A varimax rotation using Kaiser normalization also found that a five‐factor solution was the most parsimonious interpretation of the results for this 17‐item scale (Table 4). The factors explained 68.79% of the scale's variance. All of the items had factor loading after varimax rotation of >0.468. Eight items had cross‐loading. The factors analysed were as follows: *use of EBP, scientific research and analysis, sharing knowledge of evidence, sharing and use of evidence‐based guidelines* and *process of a practice change*. The first factor presented an eigenvalue of 5.719, accounting for 33.64% of the scale's variance. The four other factors had eigenvalues >1.0 (2.365, 1.447, 1.162 and 1.001, respectively), accounting for 13.91%, 8.51%, 6.84% and 5.89% of the scale's variance, respectively.

#### 4.3.2 EBP‐I scale reliability

Cronbach's alphas were computed and summed to assess whether the data from the 17 items in the German version of the EBP‐I formed a reliable scale. The overall alpha was 0.870 (95% CI = 0.833, 0.901), indicating excellent internal consistency. All the items presented a floor effect with scores near the bottom (Table 5). Cronbach's alphas for the subscales were as follows: *use of EBP* *α* = 0.841 (95% CI = 0.792, 0.881); *scientific research and analysis* *α* = 0.865 (95% CI = 0.821, 0.901); *sharing knowledge of evidence* *α* = 0.631 (95% CI = 0.501, 0.732); *sharing and use of evidence‐based guidelines* *α* = 0.639 (95% CI = 0.482, 0.748); and *process of a practice change* *α* = 0.261 (95% CI = −0.058, 0.485).

**TABLE 5:** EBP‐I scale mean, *SD*, and skewness distribution, median and interquartile (*n* = 121)

Items in the EBP‐I scale	Mean	*SD*	Skewness	Median	Interquartile 75%
Collected data on a patient problem	1.31	1.27	0.995^a^	1.00	2.00
Used evidence to change my clinical practice	1.10	1.02	1.236^a^	1.00	1.00
Shared an EBP guideline with a colleague	0.79	0.84	1.287^a^	1.00	1.00
Changed practice based on patient outcome data	0.69	1.10	1.852^a^	0.00	1.00
Evaluated a care initiative by collecting patient outcome data	0.64	1.07	1.885^a^	0.00	1.00
Informally discussed evidence from a research study with a colleague	0.57	0.75	1.618^a^	0.00	1.00
Used an EBP guideline/systematic review to change clinical practice where I work	0.57	0.86	1.905^a^	0.00	1.00
Evaluated the outcomes of a practice change	0.53	0.72	1.670^a^	0.00	1.00
Read and critically appraised a clinical research study	0.52	0.89	1.983^a^	0.00	1.00
Critically appraised evidence from a research study	0.49	0.87	2.066^a^	0.00	1.00
Promoted the use of EBP to my colleagues	0.48	0.84	2.359^a^	0.00	1.00
Shared the outcome data collected with colleagues	0.42	0.79	2.453^a^	0.00	1.00
Shared evidence from a research study with a patient/family member	0.40	0.80	2.918^a^	0.00	1.00
Shared evidence from a research study with a multidisciplinary team member	0.31	0.63	2.902^a^	0.00	0.50
Shared evidence from a study in the form of report or presentation to >2 colleagues	0.30	0.65	3.064^a^	0.00	0.00
Generated a PICO question about my clinical practice	0.28	0.61	2.926^a^	0.00	0.00
Accessed the Cochrane database of systematic reviews	0.24	0.72	3.435^a^	0.00	0.00
EBP‐I scale	9.62	8.38		7.00	14.50

^a^ Floor effect of the item.

The removal from the EBP‐I item “Accessed the National Guidelines Clearinghouse,” because no comparable database exists in Switzerland, had minimal impact on the reliability or validity of that scale's psychometric properties.

## 5 DISCUSSION

This study provides, for the first time, results on the psychometric properties of the German versions of the EBP‐B and EBP‐I scales. The results revealed that both the EBP‐B and EBP‐I scales showed good psychometric properties and are reliable. The present study's results were comparable, but not similar to those from the study by Melnyk et al. (2008).

The highest rated items concerned beliefs about the positive effects of EBP. Participants believed in the value of EBP and thought that care could be improved by implementing it. However, the lowest rated items concerned an individual's capacity to implement EBP. Participants were uncertain that they had the capacity to implement EBP and did not think that they had access to the necessary resources to do so. The highest rated EBP‐I items concerned data collection with a patient with a health problem and using proof to change clinical practice. The lowest rated items concerned the more scientific aspects of EBP: accessing the Cochrane database and formulating PICO questions only occurred rarely.

The EBP‐B scale's construct validity showed significant discontinuity between the first and second factors, with high eigenvalues confirming those in the original scale (Melnyk et al., 2008). This is an indication that, in the German‐language version, a single construct on attitudes to EBP was measured in five dimensions (*value beliefs*, *implementation beliefs*, *knowledge beliefs*, *time and difficulty beliefs,* and *effective evidence‐based care beliefs*). This was confirmed by the high factor loadings and a good Cronbach's alpha coefficient (Pett et al., 2003). All the items had a factor loading after varimax rotation of >0.4. The factor analysis revealed five cross‐loadings. Cross‐loadings are of theoretical interest when it comes to demonstrating commonalities between concepts, and they are retained if they are theoretically credible (Field, 2013), which is the case here. The cross‐loadings were also accepted and maintained in the German‐language version of the EBP‐B. The results suggest that the EBP‐B can be translated into other languages, without compromising its psychometric properties, such that the scale could be used in other international healthcare settings. Indeed, this allowed us to compare our results with those of the original scale. The results with a four‐factor solution are slightly deficient in comparison with the original scale, but they are comparable to the German‐language version's five‐factor solution. However, the item “I believe the care that I deliver is evidence‐based” had a factor loading that was clearly inferior to the original five‐factor solution.

The EBP‐I's construct validity also showed a significant gap between the first and second factors, with high eigenvalues. This indicates that, in the German‐language version, a single construct on the implementation of EBP was measured in five dimensions (*use of EBP, scientific research and analysis, sharing knowledge of evidence, sharing and use of evidence‐based guidelines* and *process of a practice change*). This was confirmed by high factor loadings and a good Cronbach's alpha coefficient (Pett, 2003). All the items had factor loadings of >0.4. The factor analysis revealed five cross‐loadings, whereas the original had had a unidimensional factor solution (Melnyk et al., 2008). This confirmed the contention that the similarities between the two concepts deserve highlighting. Thus, the cross‐loadings have been accepted and maintained in the German‐language version of the EBP‐I. Indeed, this allowed us to compare our results with those of the original scale. The results with a one‐factor solution are deficient in comparison with the original scale and to the five‐factor solution in the German‐language version.

Compared to those original scales, we found almost similar results for reliability, with a Cronbach alpha for both the German EBP‐B and EBP‐I scales exceeding 0.80. Both translated scales demonstrated high factor loading. The results, therefore, corroborated those found in previous studies, such as Verloo et al. (2017), Stokke et al. (2014), Thorsteinsson (2012), Wang et al. (2012) or Zeleníková et al. In addition, the distribution of responses varied noticeably between the two scales' items, reflecting the scales' ability to discriminate between items about beliefs or implementation.

## 6 STRENGTHS AND LIMITATIONS

This study revealed relevant information on the validity and reliability of the German‐language versions of the EBP‐B and EBP‐I scales and incorporated a sufficiently large sample of Registered Nurses from the German‐speaking parts of Switzerland. The sample corresponded to the reality in that professional field, with the majority of participants being women and an appropriate balance in terms of age and the number of years worked in the healthcare sector. Furthermore, the scales were based on the strong theoretical background developed by Melnyk et al. (2008) which have shown good validity and reliability in previous testing.

On the other hand, the sample provided us with an acceptable but non‐optimal database for a reliable factor analysis because the study was limited to the acute care department of one university hospital and so is not generalizable of all the hospitals in German‐speaking Switzerland. The representativeness of the sample could be improved by different settings. However, a direct comparison with the study by Melnyk et al. (2008) remains complicated because the sample was relatively small. Furthermore, not all the items from the original scale could be transferred to the Swiss setting.

Because study participation was voluntary, we cannot exclude the possibility that the majority of participants already held positive perceptions of EBP. Another potential influence on results is that participants self‐evaluated their knowledge of EBP, and, consequently, those data should be treated with some care.

The questionnaire was distributed, completed and returned online, and although the EBP items in both scales were marked as obligatory fields, the sociodemographic data were not. The item answers therefore contained no missing values. However, the mere fact that there were obligatory fields may have led some participants to abandon the questionnaire or just click on a random answer if, for example, they were unable or unwilling to answer a particular item. The floor effect revealed in the EBP‐I scale and the ceiling effect revealed in the EBP‐B scale may have influenced our statistics and data analysis. Furthermore, in the absence of a normal distribution, comparisons between the present results and those from other studies must remain limited. Finally, the structures of the scales were only explored; they should be tested using confirmatory factor analysis.

## 7 CONCLUSION

The translated and culturally adapted German‐language versions of the EBP‐B and EBP‐I scales enabled us to measure our target population's attitudes towards and beliefs about EBP and how far EBP is implemented in their daily clinical practice. Our findings showed that the vast majority of nurses believed in the positive effects of EBP, but that they did not regularly implement it in their daily clinical practice within a university hospital in German‐speaking Switzerland.

## 8 RELEVANCE TO CLINICAL PRACTICE AND FUTURE RESEARCH

Our findings suggest that the German‐language versions of the EBP‐B and EBP‐I scales are valid and reliable. As advocated by the ARCC model, the use of these scales to assess beliefs and implementation of EBP is an essential step to implement and sustain EBP in healthcare institutions. However, further research is needed to clarify these results as the suggested five‐factor solution for the EBP‐I's German‐language version differs significantly from the original scale's one‐factor solution.

## CONFLICT OF INTEREST

No conflict of interest has been declared by the authors.

## AUTHOR CONTRIBUTIONS

All authors: Study concept. EJ: Data collection; EJ, HV, KAP: Data analysis and interpretation. HV, KAP: Study supervision. All authors: Manuscript drafting. All authors: Contribution and revision of the manuscript.

## ETHICAL APPROVAL

The local Swiss ethical board in Bern confirmed that the study does not warrant a full ethical application and does not fall under the Swiss Federal Act on Research Involving Human Beings (CER‐BE Req‐2018‐00783). The study was on a voluntary basis for the nurses participating; all nurses were free to stop filling out the questionnaire at any time. In addition, the researchers stated that they would respect the Declaration of Helsinki's ethical principles regarding human experimentation (World Medical Association, 2013).

## Supporting information

Appendix S1

## Data Availability

The raw data set analysed in the current study is available from the corresponding author (Emmanuelle Jacquier) on reasonable request.
